# Multisystem Inflammatory Syndrome in Adults Associated With COVID-19: A Case Series on the Importance of Early Diagnosis and Corticosteroid Therapy in Prognosis

**DOI:** 10.7759/cureus.36068

**Published:** 2023-03-13

**Authors:** Anees CK, Arjun C, Bhubaneshwar Nedunchezian, Shuba Srinivasan, Anitha Theresa Augustine

**Affiliations:** 1 Department of General Medicine, Dr. Moopen's Medical College, Wayanad, IND; 2 Department of General Medicine, Sri Madhusudan Sai Institute of Medical Sciences and Research, Chikkaballapur, IND

**Keywords:** covid-19, sars-cov-2, intravenous immunoglobulins (ivig), corticosteroid treatment, mis-a

## Abstract

Multisystem inflammatory syndrome in adults (MIS-A) is a rare condition that can occur after an adult has been infected with severe acute respiratory syndrome coronavirus 2 (SARS-CoV-2). It can occur anywhere between two and 12 weeks after the beginning of acute coronavirus disease 2019 (COVID-19) infection and is characterized by extrapulmonary multiorgan failure. It is primarily seen in young and previously healthy individuals. The exact prevalence of MIS-A is unclear. It is likely underdiagnosed due to overlapping symptoms with severe COVID-19 and difficulty in identifying the syndrome without a preceding COVID-19 infection. The pathogenesis of MIS-A is also largely unknown but is likely caused by an immune response that is dysregulated or antibody-mediated. Treatment primarily involves corticosteroids, but severe cases may require intravenous immune globulin (IVIG). The timing of starting corticosteroid therapy is crucial, as delays can result in increased complications and a longer hospital stay.

## Introduction

The primary organ damaged by coronavirus disease 2019 (COVID-19) is the lungs. In contrast, its postinfectious sequelae manifest a few weeks after the initial illness, resulting in a multiorgan failure that typically spares the lungs [1]. Medical professionals across the globe have noticed a sickness showing the pattern of multisystem involvement in adults after acute COVID-19, resembling the multisystem inflammatory syndrome in children (MIS-C) after its initial discovery in early 2020 [2]. But to diagnose it as MIS-A, there are several unresolved issues. Primarily, it is unknown what is the exact incidence of MIS-A. Due to overlapping symptoms with severe COVID-19, MIS-A is probably underdiagnosed, especially in individuals with several coexisting conditions. Similar to MIS-C, there could be various MIS-A presentations among different age groups. Furthermore, difficulty in identifying MIS-A increases when the patient develops it without a significant preceding COVID-19, which may have been a mild illness resembling the common cold or even a subclinical disease, and the patient may not have sought any medical aid or tested for COVID-19 at that time [3,4]. A negative COVID-19 test at the time of diagnosis is not uncommon and can add to the complexity of diagnosis.

Although the pathogenesis of MIS-A is unclear, it is most likely caused by an immune response that is dysregulated or antibody-mediated [5]. MIS-A can present with features of fever, headache, cardiac dysfunction, skin rashes, abdominal pain, and elevated markers of inflammation. But these symptoms and signs can vary widely in the order of appearance and severity from patient to patient. Severe respiratory illness is uncommon [6]. Patients primarily benefit from corticosteroids. Severe cases may require treatment with IVIG or plasma exchange therapy [5]. Still, if the treating physicians fail to recognize this newly discovered phenomenon, which can resemble familiar illnesses like a complicated tropical fever or sepsis, they may postpone diagnosis and prompt treatment. Here, we discuss three cases of MIS-A in previously otherwise healthy young individuals, emphasizing the importance of early diagnosis and corticosteroid therapy on prognosis.

## Case presentation

Case 1

A 31-year-old gentleman complained of high-grade intermittent fever, headache, and vomiting for four days. He was diagnosed with COVID-19 three weeks ago, managed by home isolation and symptomatic treatment. There was no history of hypoxia requiring oxygen supplementation. The patient's symptoms improved significantly within one week. On examination, he was febrile and had a generalized non-itchy erythematous maculopapular rash. He also had features of conjunctivitis, such as conjunctival congestion and erythema, without obvious purulent discharge. The patient was hemodynamically stable. There was no lymphadenopathy, hepatomegaly, or splenomegaly. Respiratory system examination was normal, and the patient had normal oxygen saturation on room air.

Laboratory investigations showed an elevated total leucocyte count of 16,000 cells/µL (4000-11,000 cells/µL) and a differential count with high neutrophils (93%) and reduced lymphocytes (6%). The platelet count on admission was 96000/µL (1.5-4.5 lakh/µL). Inflammatory markers were elevated with a C-reactive protein (CRP) level of 96 mg/L (0-10 mg/L) and a serum ferritin level of 656 ng/L (24-336 ng/mL). The D-dimer value was 1325 ng/mL (<500 ng/mL). Liver function tests on admission revealed elevated serum transaminases (AST - 258 IU/L; ALT - 296 IU/L) and a normal serum bilirubin level. Renal parameters were normal. The COVID-19 reverse transcription-polymerase chain reaction (RT-PCR) and rapid antigen tests were negative, whereas COVID-19 immunoglobulin G (IgG) was positive and IgM was negative. Other blood investigations were negative, including serological tests for dengue viral fever, leptospirosis, scrub typhus, and peripheral smear for the malarial parasite. Chest X-ray and ultrasonography of the abdomen were also normal. Moreover, there was no sign of bacteremia, which was confirmed later by a negative blood and urine culture result.

**Figure 1:**
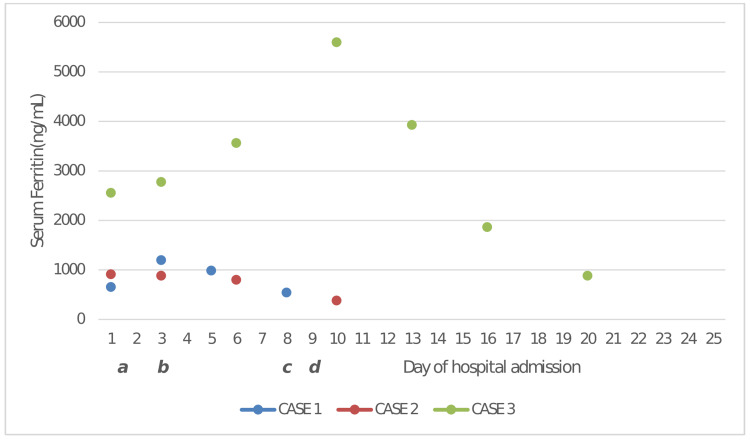
Graph showing the temporal trend of serum ferritin level (ng/mL) with the treatment of three adult patients diagnosed with MIS-A at Dr. Moopen’s Medical College, Wayanad, Kerala, India, from July 2021 to March 2022 *a*. All three patients were started on methylprednisolone within 24 hours of hospital admission after the initial laboratory results. *b*. Dose of methylprednisolone was hiked for case 2 on the third day of admission. *c*. Dose of methylprednisolone was hiked to the pulse dose regimen for case 3 on day 8 of admission. *d*. Case 3 was started on IVIG on day 9 of admission.

 In light of the recent COVID-19 infection and the unlikelihood of other illnesses, a provisional diagnosis of MIS-A was made and treatment with corticosteroids was started. We commenced with an injection of methylprednisolone 40 mg twice daily. The patient improved remarkably within 48 hours with the resolution of fever and evanescence of rashes. Laboratory parameters also showed considerable improvement, with CRP levels falling to 12 mg/L from 96 mg/L at admission. Serum ferritin also declined over five days (Figure 1). Methylprednisolone was continued orally for two weeks with a gradually tapering dose and then stopped. On follow-up after two weeks, the patient was asymptomatic, and laboratory parameters were normal.

Case 2

A 23-year-old lady patient presented with a fever for five days, vomiting, and loose stools for one day. She was diagnosed with COVID-19 around one and a half months before her current admission, which was managed symptomatically by home isolation. Her temperature on admission was 103 °F. There were features of conjunctivitis and axillary and cervical lymphadenopathy. Systemic examination revealed mild diffuse abdominal tenderness. Respiratory system examination was normal, with no signs of hemodynamic compromise.

The patient had leucocytosis with >90% neutrophils and a platelet count of 1.1 lakh/µL on initial laboratory evaluation. Inflammatory markers showed marked elevation, serum ferritin was more than 2000 µg/mL(11-307 ng/mL), and CRP was more than 192 mg/L(0-10 mg/L). The D-dimer value was 912 ng/mL (<500 ng/mL). COVID-19 RT-PCR and rapid antigen test were negative, whereas COVID-19 IgG and IgM were positive. Serological tests for dengue viral fever, leptospirosis, scrub typhus, and peripheral smear for the malarial parasite were negative, and the anti-nuclear antibody (ANA) test was also negative. Fine-needle aspiration cytology of the left cervical lymph node depicted changes consistent with reactive lymphadenopathy. Moreover, mild hepatomegaly and mild splenomegaly were observed in an ultrasound scan of the abdomen, but these changes were not clinically apparent.

The patient was treated with an injection of methylprednisolone 40 mg twice daily as the initial dose. In the absence of significant clinical or laboratory improvement, even after 48 hours, the dose of methylprednisolone was escalated to 80 mg twice daily, to which the patient responded. The patient became afebrile on the fourth day of steroid therapy, and the inflammatory markers also showed a falling trend (Figure 1). She was then discharged on a tapering dose of oral methylprednisolone for two weeks, and follow-ups were uneventful.

Case 3

A 21-year-old gentleman with no known comorbidities presented with high-grade fever and headache for nine days. His only significant history was a recent COVID-19 infection about a month ago, managed symptomatically by home isolation. He was conscious, oriented, and hemodynamically stable on examination, except for a 112 beats/minute tachycardia. His oxygen saturation was 98% at room air, and he was febrile at 104 °F. He also had a generalized maculopapular rash over his body, bilateral conjunctival congestion, and cervical lymphadenopathy.

The initial laboratory evaluation showed a total leukocyte count of 11,870 cells/µL (4000-11,000 cells/µL) with 86% neutrophils and a platelet count of 74,000/µL (1.5-4.5 lakh/µL). Inflammatory markers were markedly raised, CRP was 192 mg/L(0-10 mg/L), and ferritin was more than 2000 µg/mL(24-336 ng/mL). The D-dimer value was 625 ng/mL (<500 ng/mL). The liver functions test showed a moderate rise in serum transaminase levels with normal bilirubin. Blood urea and serum creatinine levels were normal. COVID-19 RT-PCR and rapid antigen test were negative, whereas COVID-19 IgG and IgM were positive. Serological tests for Dengue viral fever, leptospirosis, scrub typhus, and peripheral smear for the malarial parasite were negative. ANA screening test and rheumatoid arthritis (RA) factor were negative. Blood and urine cultures taken on admission showed no bacterial growth later. Given the recent COVID-19 infection and the unlikelihood of another diagnosis, the possibility of MIS-A was suspected.

The patient was started on an injection of methylprednisolone 40 mg twice daily, along with other supportive measures. However, he did not show much improvement the following day, as he had persistent high-grade fever and tachycardia. On the third day of admission, his methylprednisolone dose was increased to 80 mg twice daily. Despite this, his symptoms did not improve much, and he also started having cardiac-sounding central chest pain. Electrocardiogram showed non-specific ST-T changes. Troponin I was found to be elevated (2.3 ng/mL). An emergency bedside echocardiogram revealed mild to moderate left ventricular systolic dysfunction with no regional wall motion abnormality.

Considering rapid deterioration with progressive involvement of multiple organs, including the heart, the patients' methylprednisolone dose was hiked to a pulse dose regimen (500 mg IV once daily). His symptoms of chest discomfort continued to worsen, and he also started having breathing difficulty. He was developing pulmonary edema and hypotension secondary to left ventricular systolic dysfunction. Serial troponin I after 12 hours increased to more than 11 ng/mL, and a repeat echo showed severe left ventricular systolic dysfunction. The patient was put on mechanical ventilatory support due to significant pulmonary edema and hypotension After a multidisciplinary discussion involving a critical care specialist, cardiologist, and infectious disease specialist; he was also started on human immunoglobulin - IVIG (2 g/Kg) according to the weight and was given over three days. The patient improved in terms of hemodynamic parameters, left ventricular function, and inflammatory markers after two days of therapy with human immunoglobulin (Figure 1). Injection methylprednisolone 500 mg was given for three days and then changed to oral methylprednisolone according to weight. He was gradually weaned from mechanical ventilation and discharged from our hospital after a week. On follow-up after two weeks, the patient was functioning well, and laboratory parameters normalized.

## Discussion

MIS-A is a rare condition that occurs in some people who have previously had COVID-19. The Centers for Disease Control and Prevention (CDC) states that for a case to be considered a typical MIS-A presentation, the individual must be 21 or older and hospitalized with a severe illness, have a positive test for COVID-19 (RT-PCR, antigen, or antibodies), have severe problems with one or more organs outside of the lungs, and have high levels of inflammation in the body as evidenced by the laboratory values of inflammatory markers. This definition excludes organ problems caused by tissue hypoxia [4].

In the cases we reported, all three patients were young and previously healthy, with two being male and one female. All of them had a history of COVID-19, but it's not always the case, as sometimes the history of illness can be missed. This has been demonstrated in a review of 51 MIS-A cases, of which only 14 patients had previously clinically significant COVID-19. In the rest of the cases, the initial COVID-19 was primarily asymptomatic [3]. Patients with MIS-A tend to be young and without extensive previous health problems. Symptoms usually appear three to four weeks after a confirmed or suspected COVID-19 and include high fever, difficulty breathing, fatigue, muscle pain, abdominal pain, vomiting, diarrhea, sore throat, and a maculopapular rash. The rash is often diffuse and affects mainly the torso, upper limbs, and palms. None of our patients had respiratory system involvement or low oxygen levels during admission, but one developed cardiac dysfunction, eventually leading to hemodynamic compromise and hypoxia. Headache is a common symptom, but none of the patients was further evaluated with brain imaging or lumbar puncture. Compared to other severe cases of COVID-19, patients with MIS-A are more likely to have cardiac dysfunction. The first case series of the CDC's Morbidity and Mortality Weekly Reports (MMWR), which comprised 11 MIS-A patients, seven of whom experienced cardiogenic shock upon presentation, provides more support for this [4]. A similar study of 51 MIS-A patients revealed that cardiovascular involvement (82.4%) and gastrointestinal symptoms (72.5%) were the most commonly reported findings [3]. The demographics, clinical presentation, and treatment outcomes of all our patients have been summarized in Table 1.

**Table 1:** Clinical presentation, diagnostic tests other than laboratory studies, treatments, and outcomes of three adult patients diagnosed with MIS-A at Dr. Moopen's Medical College, Wayanad, Kerala, India, from July 2021 to March 2022 Abbreviations: Ab = antibody; IVIG = intravenous immunoglobulin; RT-PCR = reverse transcription-polymerase chain reaction; RAT = rapid antigen test; TTE = transthoracic echocardiogram; USG = ultrasonography

Case No.	Age (Years)	Sex	Clinical presentation	Pre-existing medical illness	Previous COVID-19 infection or testing	COVID-19 testing during the current admission	Imaging and other diagnostic tests	Treatments given	Outcome
1	31	Male	Fever, Headache, Maculopapular rash, Conjunctivitis	Nil	Yes	RT-PCR(-), RAT(-), IgG Ab(+), IgM Ab(-)	TTE: Normal Chest X-Ray: Normal USG Abdomen - normal	Corticosteroids	Discharged after 8 days
2	23	Female	Fever, Vomiting, Diarrhoea, Lymphadenopathy, Conjunctivitis	Nil	Yes	RT-PCR(-), RAT(-), IgG Ab(+), IgM Ab(+)	TTE: Normal Chest X-Ray: Normal USG Abdomen – mild hepato-splenomegaly	Corticosteroids	Discharged after 11 days
3	21	Male	Fever, Headache, Maculopapular rash, Lymphadenopathy	Nil	Yes	RT-PCR(-), RAT(-), IgG Ab(+), IgM Ab (+)	TTE: Severe left ventricular systolic dysfunction Chest X-ray: features of pulmonary edema USG Abdomen - normal	Corticosteroids, Antibiotics, IVIG	Discharged after 22 days

There are many case reports on MIS-A from different parts of the world, and systematic reviews of these reports are available now [7]. Like our cases, many patients had been relatively asymptomatic when they first contracted COVID-19. Blood tests on these patients showed high levels of inflammation during hospitalization with MIS-A compared to the patient's primary COVID-19. All patients in our study had elevated inflammatory markers on admission, which showed rapid improvement with corticosteroid therapy. Troponin I and serum creatinine values showed an abnormality only in case number three, which presented late. The values of various laboratory parameters during admission and peak values during the hospital's course have been summarized in Table 2.

**Table 2:** Laboratory study results (on admission, peak value during the course in the hospital, and on discharge) of the three adult patients diagnosed with MIS-A at Dr. Moopen’s Medical College, Wayanad, Kerala, India, from July 2021 to March 2022 Abbreviations: ALT = alanine aminotransferase; AST = aspartate aminotransferase; CRP = C-reactive protein; IL-6 = interleukin-6 * Normal ranges of laboratory values: Total leukocyte count 4000–11000 cells/μL; CRP 0–10 mg/L; D-dimer <500 ng/mL; Ferritin 24–336 ng/mL (men), 11–307 ng/mL (women); IL-6 ≤3.4 pg/mL; ALT 7–55 IU/L; AST 8-48 IU/L; Troponin I <0.01 ng/mL; Serum creatinine 0.7-1.3 mg/dL Abbreviations: ALT = alanine transaminase; AST = aspartate aminotransferase

Laboratory Investigations	Case 1			Case 2			Case 3		
	On Admission	Peak*	On Discharge	On Admission	Peak*	On Discharge	On Admission	Peak*	On Discharge
Total Leukocyte Count (cells/μL)	16080	22060	12,000	14320	18420	10,876	11870	28056	14,876
CRP (mg/L)	96	>192	48	192	>192	12	>192	>192	96
D-dimer (ng/mL)	1325	2400	482	2000	2560	866	688	1860	1092
Ferritin (ng/L)	656	1200	544	912	912	384	2560	5600	886
IL-6 (pg/mL)	21.2	146.4	10.8	64.8	96.7	23.9	112.6	112.6	43.9
Troponin – I (ng/mL)	< 0.01	<0.01	<0.01	<0.01	<0.01	<0.01	0.5	11	1.2
ALT (IU/L)	293	326	96	126	456	56	354	2360	224
AST (IU/L)	258	296	78	98	378	74	299	1884	198
Serum Creatinine (mg/dL)	0.7	1.1	0.9	0.9	0.9	0.6	0.8	1.5	1.2

Many recommendations extrapolate from MIS-C data to help care for patients with MIS-A but stress that this method of managing MIS-A has not been tested. Currently, corticosteroids, IVIG, and supportive care are proposed therapies for MIS-A in the medical literature [1]. Out of our three patients, two were cured entirely with non-pulsing doses of corticosteroids alone. At the same time, one patient (case number three) who presented late responded poorly to initial lower doses of steroids and had more complications than the others who presented within one week of the symptom's onset. The patient who presented late had clinical and laboratory parameters comparable with the others on presentation, but further progress of the illness and eventually requiring mechanical ventilatory support was unexpected. However, this patient (case number three) responded dramatically to combined therapy with a pulse dose of methylprednisolone and IVIG. One of the important observations we made is that the timing of initiation of corticosteroid therapy could be a deciding element in the patient's response to treatment and overall prognosis.

The CDC recommends the COVID-19 vaccine as the best protection against MIS-A based on current understanding. However, there are no data on the safety and effectiveness of COVID-19 vaccines in individuals with a history of MIS-C or MIS-A. This recommendation on the efficacy of the COVID-19 vaccine in preventing MIS-A becomes more complicated when case reports show the incidence of MIS-A as a post-vaccine complication [8]. All our cases had received two doses of the COVID-19 vaccine, and the second dose was at least three months before the hospitalization for MIS-A.

We suggest that doctors should always consider MIS-A as a differential diagnosis in patients with evidence of multisystem inflammatory illness and severe extrapulmonary organ dysfunction occurring within two to 12 weeks of COVID-19.

## Conclusions

A high index of suspicion may help diagnose MIS-A in adults presenting with multisystem inflammatory manifestations following COVID-19. Early initiation of systemic corticosteroids may be lifesaving, although empiric evidence for this is lacking due to the paucity of randomized controlled trials. In severe cases, IV immunoglobulins may hasten recovery and augment the anti-inflammatory action of systemic steroids. To more clearly describe clinical entities like MIS-A, as well as to decrease the selection bias, large data registries and clinical cohorts are required. Furthermore, a better understanding of pathogenesis and genetic association may help identify people at risk of developing post-viral inflammatory syndromes like MIS-A. Additionally, more investigation is needed to determine why MIS-A affects men more commonly.
